# Aerobic Sludge Granulation in a Full-Scale Sequencing Batch Reactor

**DOI:** 10.1155/2014/268789

**Published:** 2014-04-15

**Authors:** Jun Li, Li-Bin Ding, Ang Cai, Guo-Xian Huang, Harald Horn

**Affiliations:** ^1^Department of Municipal Engineering, Zhejiang University of Technology, No. 18 Chao Wang Road, Hangzhou 310014, China; ^2^Yancang Wastewater Treatment Plant, Haining 314422, China; ^3^Water Chemistry and Water Technology, Karlsruhe Institute of Technology, Engler-Bunte-Ring 1, 76173 Karlsruhe, Germany

## Abstract

Aerobic granulation of activated sludge was successfully achieved in a full-scale sequencing batch reactor (SBR) with 50,000 m^3^ d^−1^ for treating a town's wastewater. After operation for 337 days, in this full-scale SBR, aerobic granules with an average SVI_30_ of 47.1 mL g^−1^, diameter of 0.5 mm, and settling velocity of 42 m h^−1^ were obtained. Compared to an anaerobic/oxic plug flow (A/O) reactor and an oxidation ditch (OD) being operated in this wastewater treatment plant, the sludge from full-scale SBR has more compact structure and excellent settling ability. Denaturing gradient gel electrophoresis (DGGE) analysis indicated that *Flavobacterium* sp., uncultured beta proteobacterium, uncultured *Aquabacterium* sp., and uncultured *Leptothrix* sp. were just dominant in SBR, whereas uncultured bacteroidetes were only found in A/O and OD. Three kinds of sludge had a high content of protein in extracellular polymeric substances (EPS). X-ray fluorescence (XRF) analysis revealed that metal ions and some inorganics from raw wastewater precipitated in sludge acted as core to enhance granulation. Raw wastewater characteristics had a positive effect on the granule formation, but the SBR mode operating with periodic feast-famine, shorter settling time, and no return sludge pump played a crucial role in aerobic sludge granulation.

## 1. Introduction

Aerobic granulation is a novel and promising technology for wastewater treatment [1–3]. Aerobic granular sludge is formed by microbial self-aggregation and has advantages such as excellent settling ability, dense and strong microbial structure, high biomass retention, ability to withstand a high organic loading rate and tolerance to toxicity compared with conventional activated sludge [4–7]. However, the operational conditions of aerobic granular sludge are strictly limited by factors like reactor configuration, substrate composition, selecting pressure, volume exchange ratio, hydrodynamic shear force, organic loading rate (OLR), feast-famine regime, feeding strategy and cycle time [8, 9]. To date, it still lacks in theoretical models to explain the mechanism of aerobic granulation although several hypotheses and mathematic models have been proposed [10]. Furthermore, most of the previous studies on aerobic granulation were carried out in laboratory-scale reactors [5, 9] and only a few were done in pilot-scale reactors [11–14]. In addition, there is very limited literature available although several full-scale plants have been built in the Netherlands, Portugal and South Africa [15, 16].

The world's first pilot-scale aerobic granular sludge reactor for real wastewater treatment was started up in September 2003 in Ede, the Netherlands, consisting of two parallel biological reactors with a height and diameter of 6 m and 0.6 m, respectively [11]. An SBR with a working volume of 1 m^3^ and a diameter of 0.5 m for treating low-strength wastewater in a wastewater treatment plant (WWTP) in China [12] and another SBR with a working volume of 0.1 m^3^ and a diameter of 0.25 m fed with synthetic wastewater in Spain [14] were built up. Aerobic granules were successfully cultivated in all these previous pilot-scale systems. The results showed that reasonable organic loading rate, high H/D ratio, sequencing batch operation and settling time still could be necessary factors. A recent study showed that an intensive anaerobic contact of granules and easily degradable organic carbon at the beginning of each SBR cycle stabilize granular growth, phosphorus and nitrogen removal [17].

Gansbaai WWTP was reported to be the first full-scale domestic sewage treatment work in the world using aerobic granular sludge technology in an upgrade project [15, 18]. It was designed for 4,000 m^3^ d^−1^ of high strength septic influent consisting of three parallel reactors with a height of 7 m and diameter of 18 m. Another full-scale SBR in Epe, the Netherlands, was designed for 59,000 person equivalents and treating up to 1,500 m^3^ h^−1^ municipal wastewater with a high contribution of industrial waste from slaughterhouses [15]. Nevertheless, the detailed data of the operational performance of full-scale applications have not been presented.

Yancang WWTP was located in Haining, a coastal city in Eastern China. Attention has been paid because some small particles were observed in activated sludge from both anaerobic/oxic plug flow process (A/O) and oxidation ditch process (OD) in this plant since 2008. Particularly, about 60–79 mL g^−1^ of SVI indicated that the sludge from these two continuous flow reactors had better settling ability compared with normal activated sludge.

The major aim of this work was to demonstrate the feasibility of cultivating aerobic granular sludge in an SBR, particularly for full-scale application. Successively, a lab-scale SBR, a pilot-scale SBR and a full-scale SBR were set up and used for the treatment of this wastewater through the development of aerobic granular sludge. The characteristics of different sludges from A/O, OD and SBR were compared. The main factors for aerobic sludge granulation in this full-scale SBR were discussed.

## 2. Material and Methods

### 2.1. Wastewater and Inoculating Sludge

Wastewater in Yancang WWTP included approximately 30% domestic sewage and 70% industrial wastewater from printing and dyeing, chemical, textile and beverage. The characteristics of the wastewater were showed as follows: chemical oxygen demand (COD_Cr_) of 200–600 mg L^−1^, biochemical oxygen demand (BOD) of 50–105 mg L^−1^, ammonium nitrogen (NH_4_^+^-N) of 28.0–40.0 mg L^−1^, total phosphorus (TP) of 2.0–4.0 mg L^−1^ and temperature of 18–30°C. The average influent BOD/COD ratio was only about 0.23 which belonged to bio-refractory wastewater. The lab-scale, pilot-scale and full-scale SBR were all introduced with the same raw wastewater and inoculated sludge from the second sedimentation tank in OD.

### 2.2. The A/O Set up and Operation

**Figure 1:**
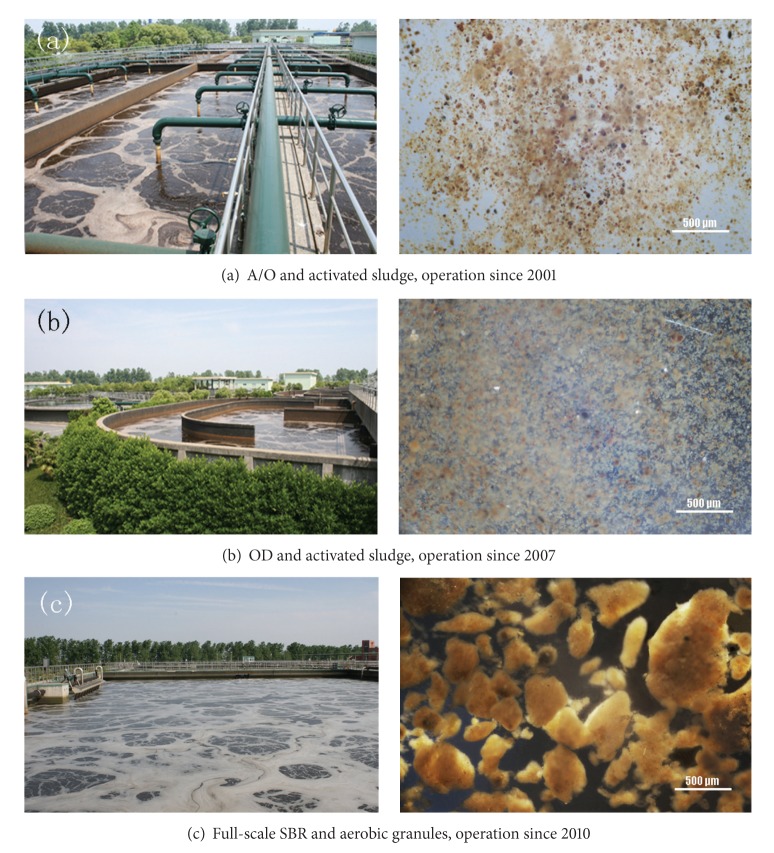
Photographs of reactors and sludge of A/O, OD and full-scale SBR in Yancang WWTP.

The A/O process was built and came into operation in 2001 treating 10,000 m^3^ per day (Figure 1(a)). It included a regulation tank, reaction tank, primary sedimentation tank, A/O tank, second sedimentation tank, oxidation contact tank and final sedimentation tank. The A/O plug flow process in this WWTP was designed and operated in a traditional way. The second sedimentation tank was separately built with the main bioreactor and the returned sludge was pumped from second sedimentation tank to anaerobic zone. It include an bioreactor consisted of an anaerobic zone with a size of 25 m length, 25 m width and 5 m depth followed by an oxic zone with a size of 55 m length, 25 m width and 5 m depth.

### 2.3. The OD Set up and Operation

In order to meet the treating requirements of the increasing wastewater, the second stage project of the OD process with a treating capacity of 50,000 m^3^ d^−1^ came into operation in 2006 (Figure 1(b)). It included a regulation tank, primary sedimentation tank, hydrolysis acidification tank, aerobic tank, second sedimentation tank and final sedimentation tank. The aerobic tank had a size of 90 m length, 35 m width and 3.5 m depth. The OD was designed and operated in a traditional way. The second sedimentation tank was separately built with the main bioreactor of OD. The returned sludge from second sedimentation tank was pumped to the main bioreactor of OD.

### 2.4. Lab-Scale SBR Set up and Operation

**Figure 2:**
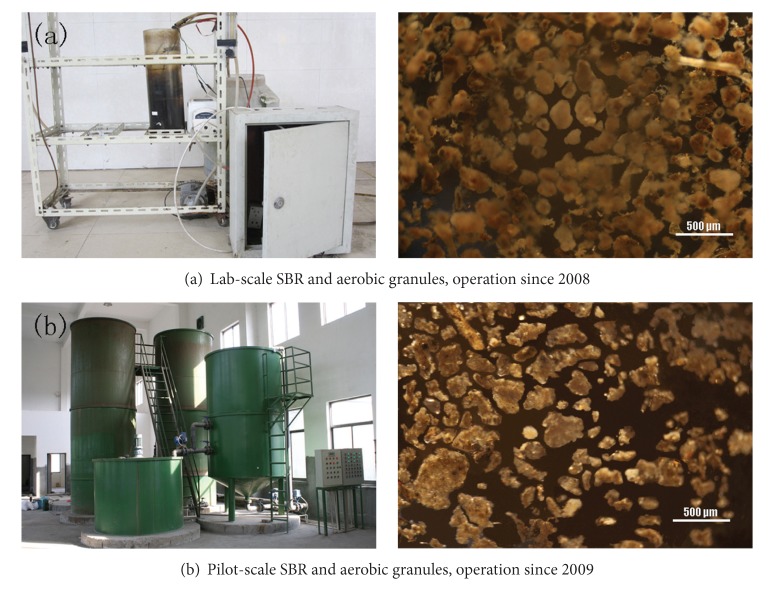
Photographs of reactors and sludge of lab-scale SBR and pilot-scale SBR in Yancang WWTP.

A column type lab-scale SBR with an H/D ratio of 2.5, working volume of 5.0 L and volumetric exchange ratio of 50% was set up in 2008 (Figure 2(a)). It was reported that Kong had successfully developed aerobic granules in four SBRs with different H/D ratio of 24, 16, 8 and 4, respectively and a higher reactor H/D ratio such as 20–30 was mostly used in literature to meet the requirement of the minimal settling velocity for granule formation [19]. So here, we defined that the reactor with an H/D below 4 was considered to be low. The raw wastewater was introduced from top of the reactor with a volume of 2.5 L per cycle. The lab-scale reactor was aerated by using a fine bubble aerator and operated in a fill-draw mode. After inoculation, the SBR was initially operated in successive cycles of 4 h, and each cycle consisted of 1 min of filling, 120 min of aeration, 60 min of settling, 20 min of effluent withdraw and 39 min of idling. After 10 days, the SBR was operated in a cycle of 4 h, which consisted of 1 minute of filling, 180 min of aeration, 10 min of settling, 20 min of effluent withdraw and 29 min of idling (Table 1). The organic loading rate (OLR) of the influent was controlled at 3.9–4.5 kg COD m^−3^ d^−1^ and superficial air velocity was controlled at 1.3 cm s^−1^. A programmable logic controller (PLC) controlled the implementation of the pumps, valves and the length of every operational batch cycle.

### 2.5. Pilot-Scale SBR System Set up and Operation

A grit chamber, a service tank and two parallel columns constituting the pilot-scale SBR system was set up in 2009 (Figure 2(b)). The two parallel columns had a height of 6 m, an internal diameter of 2 m, H/D of 2.5 and a maximum operating flow rate of 120 m^3^ d^−1^. Fine bubble aerators were used in this pilot-scale SBR. The operation was controlled by a PLC. The pilot-scale SBR system would operate in this way: the raw wastewater was pumped into the grit chamber firstly, and then it flowed into a service tank controlled by electric butterfly valve and level controller. When came to the feeding period, wastewater was pumped into two parallel columns. The wastewater was introduced from the bottom of the pilot-scale reactor. The reactor with a volumetric exchange ratio of 50% was operated in a fill-draw mode. The first stage consisted of 40 min of influent addition, 120 min of aeration, 60 min of settling and 20 min of effluent withdraw. After 7 days of operation, it came to the second stage which consisted of 40 min of influent addition, 120 min of aeration, 20 min of settling and 20 min of effluent withdraw (Table 1).

### 2.6. The Full-Scale SBR and Operation

With the expansion of service area and urbanization, an A/O with a treating capacity of 10,000 m^3^ d^−1^ constructed in 1999 and an OD with a treating capacity of 50,000 m^3^ d^−1^ constructed in 2006 did not match with the increasing wastewater. A new wastewater treatment work with a treating capacity of 50,000 m^3^ d^−1^ was required in this plant. The aims of building the third stage project in Yancang WWTP were not only to improve the treatment ability but also to meet the strict wastewater discharging standard in China. Consequently, selecting a more effective and economic process for treating wastewater was an urgent issue. Activated sludge with an SVI of 60–79 mL g^−1^ and some small granules with the size of 30–80 *μ*m existing in the A/O and OD process enlightened us whether it was feasible to cultivate aerobic granular sludge with SBR process for treating wastewater in Yancang WWTP. The A/O plug flow and OD were typical activated sludge process. The only difference was in raw wastewater compared with other WWTP. It implied that raw wastewater was the key factor for the formation of these aggregates in typical A/O plug flow and OD process. If operated in an SBR mode with certain selecting pressure, aerobic granules would be easily formed. Hence, a series of lab-scale and pilot-scale experiments were carried out and proved the feasibility of developing aerobic granules in this treatment plant. After lab-scale and pilot-scale experiments for successful aerobic granulation, the third stage project with a SBR process was built and came into operation in 2010 (Figure 1(c)) and the application of aerobic granular sludge was further studied in this work.

The full-scale SBR was divided into four separated tanks for alternative operating. Each tank had an H/D of 0.09, a volume of 12,540 m^3^ with length of 55 m, width of 38 m and depth of 6 m, respectively. Before raw wastewater flowed into full-scale SBR, it would flow into regulating reservoir, primary sedimentation tank and hydrolysis tank in turn. The effluent quality from full-scale SBR was enhanced by coagulating sedimentation. The wastewater was introduced from top of the full-scale reactor. The full-scale SBR with volumetric exchange ratio of 50%–70% was operated in a fill-draw mode. At the end of 2010, the full-scale SBR was constructed and came into operation. After inoculation, each cycle consisted of 40 min of filling, about 240 min of aeration, 60 min of settling and 30 min of effluent withdraw. After 25 days, the operation cycle consisted of 40 min of filling, about 240 min of aeration, 40 min of settling and 30 min of effluent withdraw. Six months later, the operation cycle consisted of 40 min of filling, about 240 min of aeration, 50 min of settling and 30 min of effluent withdraw (Table 1). Accurate aeration time was controlled by an intelligent system depending on variation of dissolved oxygen.

### 2.7. Analytical Methods

COD_Cr_, NH_4_^+^-N, nitrite (NO_2_^−^-N), nitrate (NO_3_^−^-N), sludge volume index with 30 min settling time (SVI_30_), mixed liquor suspended solids (MLSS) and mixed liquor volatile suspended solids (MLVSS) were analyzed in accordance to the Standard Methods [20]. Biological Oxygen Demand (BOD_5_) was measured using the WTW-OxiTop system. The morphology of sludge was observed by an Olympus CX31 microscope and a digital camera (Canon EOS 30D). The size of granules was analyzed by an image analysis system (Image-Pro Plus 6.0, Media Cybernetics). The element distribution of raw wastewater and granules was analyzed by X-ray fluorescence (XRF). The toxic organic substance in raw wastewater was measured by gas chromatography/mass spectrometry (GC/MS) (SHIMADZU GCMS-QP2010). The extracellular polymeric substances (EPS) of granules were extracted using the EDTA extraction method [21]. The carbohydrate concentration in EPS was determined as glucose equivalent using enthrone-sulfuric acid method [22], whereas the protein concentration was measured as bovine albumin equivalent using the Lowry method [23].

### 2.8. DNA Extraction, Polymerase Chain Reaction (PCR) Amplification of Bacterial 16s RNA Gene and Denaturing Gradient Gel Electrophoresis (DGGE)

The sludge taken from the reactors was washed twice with PBS buffer. After the samples were centrifuged at 12000 rpm for 10 min, the sludge was immersed into 588 *μ*L extraction buffer (0.1 M Tris-HCl, 0.1 M Na_2_-EDTA, 0.1 M sodium phosphate, 1.5 M NaCl) and stored in −20°C freezer until DNA extraction. Bead-beating was applied to produce a suspension of bacterial cells from sludge. Total DNA was extracted with lysozyme, proteinase K, and sodium dodecyl sulfate treatment, followed by phenol/chloroform/isoamyl alcohol (25 : 24 : 1) extraction and isopropanol precipitation.

20 ng DNA was amplified using PCR primers P2 and P3 with 40 bases of a GC clamp [24]. PCR conditions and thermal programs for DGGE have been previously described [25]. The PCR-amplified fragments were separated by DGGE using a DCode universal mutation detection system (Bio-Rad Laboratories) as described previously [26]. Quantity One 4.6.2 software (Bio-Rad) was used for band pattern analysis, which was performed by setting background subtraction at 10 using the rolling disk method. The intensity of bands was excluded from analysis which were smaller than 0.05.

Obtained sequences of DGGE bands were submitted to the GenBank database with assigned accession numbers of KF234427-KF234446 and KF273869, and then compared to the database using the Basic Local Alignment Search Tool-nucleotide (BLASTn) algorithm via http://www.ncbi.nlm.nih.gov/ for sequence identification. Sequence alignments of 16S rRNA partial sequences of reference microbes from Genbank were performed by CLC sequence Viewer software (Version 6.8). DGGE profiles were analyzed by BandScan software (Version 5.0). Microbial diversity was calculated by the Shannon diversity index (*H*) [27]. Community similarities of DGGE profiles were compared using pairwise similarity coefficients (*C*_*s*_) [28].

## 3. Results and Discussion

### 3.1. Formation and Characteristics of the Aerobic Granules in Lab-Scale SBR and Pilot-Scale SBR

In the lab-scale SBR, small granules with irregular and loose structure became visible about 20 days after inoculation. After operating for 43 days, aerobic granules with a mean diameter of 0.3 mm were dominant in the reactor. The MLSS and SVI in reactor were 6200 mg L^−1^ and 38 mL g^−1^, respectively. The MLVSS/MLSS ratio increased continuously from 51% of the inoculating sludge to 70% of the granules. The average COD removal efficiency was kept at 87.5% while NH_4_^+^-N and TP were stabled at 97% and 86% respectively. The result indicated that a low H/D ratio of the reactor was not the necessary condition for aerobic sludge granulation.

In the pilot-scale SBR, small granules were observed after 7 days of operation. After operating for 50 days, aerobic granules with a mean diameter of 0.28 mm were dominant in the reactor. The MLSS and SVI of mature granular sludge were 7500 mg L^−1^ and 43 mL g^−1^, respectively. The average COD removal efficiency in two column reactors was stabled at 88% while NH_4_^+^-N was all removed. The result implied the feasibility of application of aerobic granular sludge in full-scale SBR in this plant.

Sludges from lab-scale SBR and pilot-scale SBR were photographed after operating for 43 days and 50 days, respectively (Figure 2). They showed similar dense structure, sharp and irregular outline.

### 3.2. Formation of Aerobic Granules in Full-Scale SBR and Comparison of Sludges from A/O, OD and Full-Scale SBR

**Figure 3:**
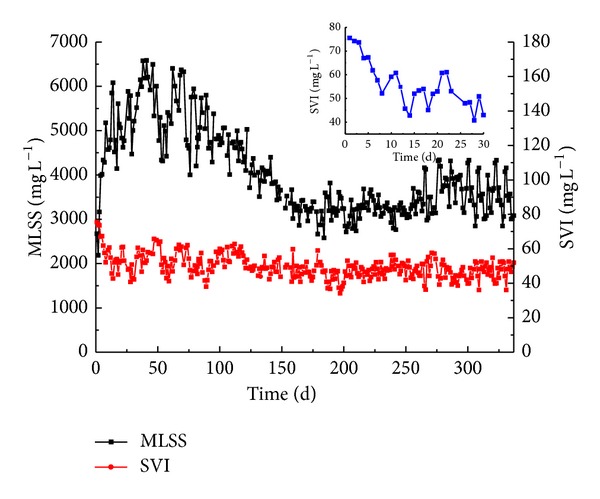
The MLSS and SVI_30_ of the sludge in full-scale SBR in Yancang WWTP from October, 2010 of the seeding sludge to September, 2011 of the granular sludge.

After inoculation in the full-scale SBR, the SVI decreased from 75.5 mL g^−1^ of the inoculated sludge to 43 mL g^−1^ after 14 days operation. Then the SVI increased to 61 mL g^−1^ on day 21 and fell down to 43 mL g^−1^ on day 30. In the next 310 days, the average SVI was stabled at 48 mL g^−1^ (Figure 3). Accordingly, the MLSS increased up to 6583 mg L^−1^ in day 38 with the decreasing of SVI. Then, the MLSS decreased gradually and stabilized at approximately 3600 mL g^−1^ due to discharging a certain amount of sludge every day including granules and some flocs (Figure 3).

The average SVI in the A/O and OD process were 61.1 mL g^−1^ and 60.7 mL g^−1^ respectively (Table 2), which showed better settling ability compared with conventional activated sludge. The average MLSS concentrations and MLVSS to MLSS ratio in the A/O and OD process were 5471 mg L^−1^, 5818 mg L^−1^ and 51.0%, 57.2%, respectively. The sludge in the A/O and OD process showed good settling ability due to relatively high inorganic proportions existing in activated sludge. The removal performance in A/O and OD were kept well with NH_4_^+^-N removal rate of 94.3% and 94.8% and COD removal rate of 82.2% and 74.0%, respectively. However, the TN removal rate in full-scale SBR was 59.6% which was much higher compared with A/O and OD due to simultaneous nitrification and denitrification effect of aeobic granules. TP only decreased from 2.5 mg/L to 1.2 mg/L in full-scale SBR since there was no anaerobic phase existed and the removal of TP was mainly depend on post-treatment of physical and chemical. Granules in the full-scale SBR showed the best settling ability and the highest settling velocity among these three processes after granulation (Table 2). It indicates that aerobic granular sludge in the SBR process is advantageous compared to traditional activated sludge in the A/O and OD process. A much higher settling velocity of aerobic granules allowed for less settling time in the SBR which in turn improved the wastewater treatment efficiency in the SBR. Furthermore, a short settling time favored the growth of rapidly-settling bioparticles whereas the bioparticles with a poor settling ability were washed out [12].

Through microscope and digital camera analysis, it could be found that small granules existed in activated sludge in both A/O and OD. The A/O operated in a plug flow way while in the OD the wastewater was completely mixed. Hence, the substrate concentration in the A/O experienced a change from high to low along the reactor length which matched the feast-famine regime while in the OD the substrate concentration had almost not changed. Furthermore, the secondary sedimentation tank with a long settling time (2.5 hours) could not offer enough selecting pressure to wash out the poorly settleable sludge. In addition, the aeration tank in the A/O and OD process was separated from the second sedimentation tank. Part of the settled sludge in the second sedimentation tank was pumped back to the aeration tank for recirculation. In this way, the granule was easily destroyed by the pump. In conclusion, it was tough to cultivate aerobic granules in the A/O and OD.

### 3.3. DGGE Profile and Bacterial Community Analysis

#### 3.3.1. DGGE Profile and Phylogenetic Analysis

**Figure 4:**
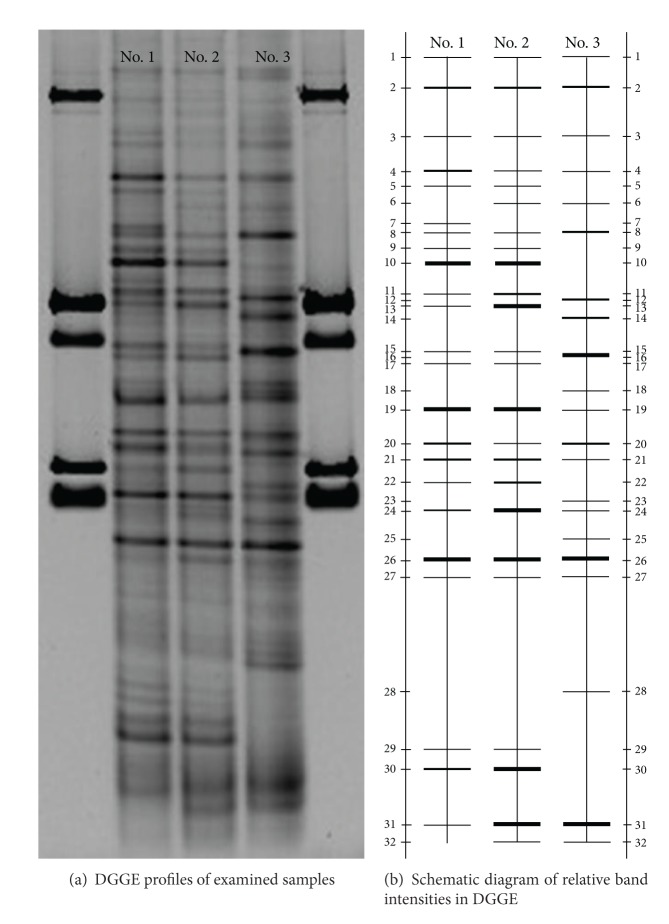
The DGGE profiles of bacterial communities in three different reactors and all sludge samples were collected on September 8, 2011. 1#: sludge from A/O; 2#: sludge from OD; 3#: sludge from full-scale SBR.

DGGE profiles (Figure 4) and similarity coefficient analysis (Table 3) indicated that under continuous flow (A/O and OD) and SBR conditions, the bacterial community composition especially for bacterial species showed a remarkable difference. Most of the bacterial species in activated sludge in A/O and OD were similar, but a noticeable difference occurred in mature aerobic granules in the full-scale SBR. It had been previously reported that the influence of operation conditions and reactor format on the community diversity was evidenced by the change in band patterns [29].

Schematic band intensities for DGGE profiles were obtained using BanScan software (Figure 4(b)). There were 32 obvious bands in DGGE profiles with 21–24 bands in every sample (Figure 4), and there was no obvious difference in the number of bands and the diversity index among the samples (Table 4). As measured, similarity coefficients of 1# and 2# were relatively high (Table 3). Some bands, such as bands 1, 2, 3, 4, 8, 19, 20, 21, 24, 26, 27 and 31 were found in all of the samples under three different processes. However, the relative intensities of the same band in different sample profiles were different. Besides, some special bands appeared in the DGGE profiles of different samples such as band 14, 16, 18 and 20, which only existed in 3# (Figure 4(b)).

Typical bands from the DGGE profiles were separated, re-amplified, sequenced and thirteen sequences were obtained. Nucleotide sequences and the abundance of sequenced DGGE bands were compared (Table 5). Sequence analysis showed that bands 10, 12, 16 and 26 had similarity levels of 100%, 99%, 100% and 100%, respectively, which was more than the genus similarity level [30]. It was therefore considered that sequences of these four bands were from* Nitrospira*,* Flavobacterium*,* Aquabacterium *and* Thauera* genus, respectively. According to the DGGE profiles,* Flavobacterium* (band 12) dominated in the granules from the full-scale SBR although it was not the dominant group in the initial sludge from the OD used as seed sludge. It was reported that* Flavobacterium* is a significant genera of floc-forming bacteria [31] producing extracellular polymers to bind cells together. Earlier works also proved that* Flavobacterium *was dominant in granules from the full-scale SBR while not dominant in seed sludge [32]. It was interesting to note that Bacteroidetes (band 13) was dominant in A/O and OD but did not exist in the granular sludge in the full-scale SBR. Former works had proved that the Bacteroidetes bacterium was washed out at short settling times and did not contribute to sludge granulation [33]. In our study, the Bacteroidetes bacterium was washed out under SBR operational conditions, but retained under A/O and OD operational conditions due to the longer settling time.* Nitrospira *(band 10) dominated in A/O and OD but not in the full-scale SBR after granulation.* Thauera *(band 26) was present significantly in all three reactors and it played an important role in nitrogen removal [33].

### 3.4. Main Factors for Aerobic Granulation in Full-Scale SBR

#### 3.4.1. The Effect of Raw Wastewater on Aerobic Granulation

Excellent performance of aerobic granules in the lab-scale SBR, pilot-scale SBR, and particularly full-scale SBR suggested a necessary discussion about the role of raw wastewater in aerobic granulation.

**Figure 5:**
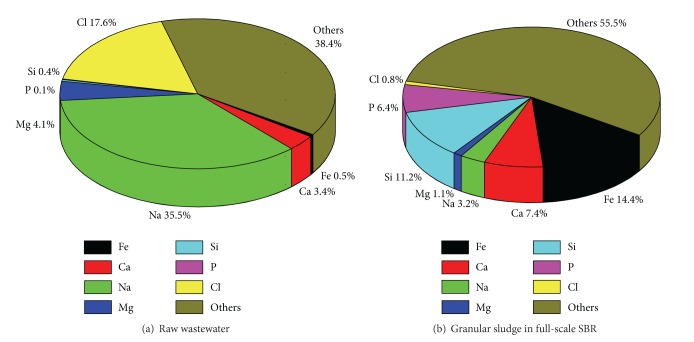
Element analysis of raw wastewater and granular sludge in full-scale SBR by X-ray fluorescence (XRF).

Chemical elements in raw wastewater and granules from the full scale SBR were analyzed by XRF (Figure 5). The contents of Na and Cl were 35.43% and 17.59% respectively in raw wastewater due to the fact that this WWTP is located at the seaside and is treating some industrial wastewater. It was reported that the presence of salt in the treated effluent did not cause a detrimental effect on the operation of the reactor once the aerobic granules were formed [34] or the granular structure was stable throughout the whole experimental period when subjected to different salinity [35]. It indicated that Fe, Si, Ca and P were precipitated in aerobic granules since the contents of these elements in granules were higher than those in raw wastewater. It had been demonstrated that the presence of divalent and trivalent mental ions could act as a bridge between negatively charged groups on the cell surface which was important in the aggregation progress [36, 37]. The effect of Ca^2+^ and Mg^2+^ enhancing the sludge granulation in the SBR was widely recognized [38–40]. Some studies indicated that Al and Fe were necessary in the development of compact aerobic granules structure with excellent settling properties [41, 42]. Si was also precipitated significantly in granules; it had been reported that Si laid the foundations for the aerobic granule structure and supported the strength of matured granules [41].

**Figure 6:**
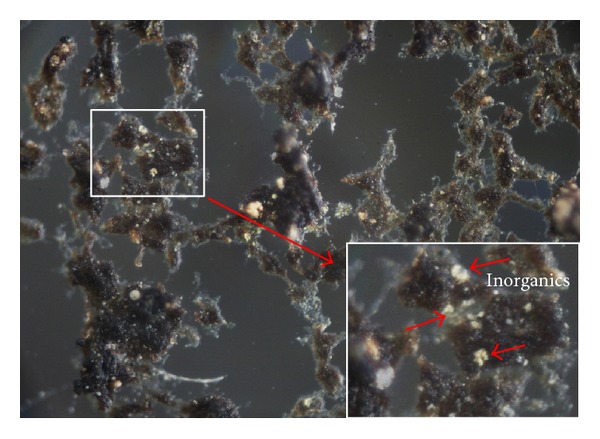
Image of dry aerobic granular sludge from full-scale SBR. Some inorganics act as cores in granules showed by short arrows.

Large amounts of inorganics composed of Fe, Si, Ca and P existing in the raw wastewater obviously provided nuclei to accelerate microbial aggregation. These inorganic solids were found in aerobic granules from this full-scale SBR (Figure 6). Huishoff used hydro-anthracite as an additional inert support particle accelerating the anaerobic granulation [43]. Granular activated carbon (GAC) was also added for sludge granulation in the SBR with low-strength wastewater [44]. Powdered activated carbon (PAC) and GAC were added during the start-up of upflow anaerobic sludge blanket (UASB) to accelerate granulation [45]. However, these inorganics also caused low VSS/SS ratio in sludge.

**Figure 7:**
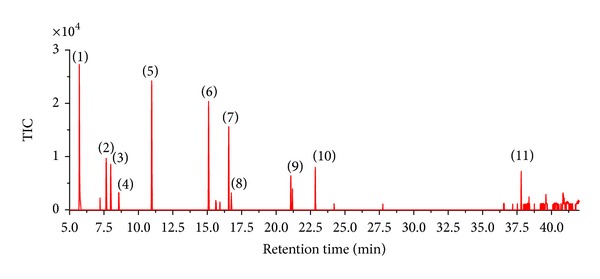
Volatile and semi-volatile organic compounds in raw wastewater by GC/MS analysis: (1) 2-Bromo-2-nitropropane (2) Propanoic acid, 2-hydroxy-, 2-methylpropyl ester (3) Formic acid, 2-propenyl ester (4) Aniline (5) Anline, N-methyl- (6) Isoquinoline (7) 4-Aminoheptane (8) 1,2,4-Thiadiazole, 5-amino-3-propyl- (9) Phenol, 3,5-bis(1,1-dimethylethyl)- (10) Pyrimidine-2,4(1H,3H)-dione, 5-amino-6-nitroso (11) Oxalic acid, isobutyl propyl ester.

It was believed that the formation and stability of aerobic granules were closely related to the sludge EPS [46]. Therefore, the EPS content of sludge in A/O, OD and full-scale SBR were analyzed respectively (Table 2). The presence of high PN contents in these three different sludges indicated the probable effect of raw wastewater in WWTP, which contained bio-refractory or toxic compounds such as Benzenamine, Benzenamine N-methyl- and Isoquinoline (Figure 7). Previous studies implied that the EPS production, especially the protein components of microbes, increased due to the exposure to toxic environment [47]. The PS content in the full-scale SBR of 20.8 mg g^−1^ VSS was higher than in the A/O (15.2 mg g^−1^ VSS and OD of 13.0 mg g^−1^ VSS). PS could mediate both cohesion and adhesion of cells, and played an important role in maintaining the structure and stability of aerobic granules [48].

#### 3.4.2. The Effect of Operating Mode on Aerobic Granulation

**Figure 8:**
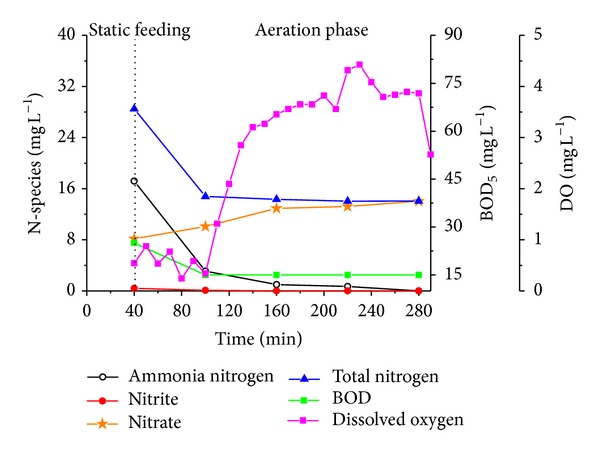
Profiles of NH_4_^+^-N, NO_2_^−^-N, NO_3_^−^-N, TN, DO and BOD concentrations with in a representative cycle in full-scale SBR. Note the influent with NH_4_^+^-N of 28.2 mg L^−1^, TN of 34.5 mg L^−1^, BOD_5_ of 85 mg L^−1^.

Comparing the operating mode of SBR with A/O and OD, the unique feature of the full-scale SBR was its cyclic operation mode which would lead to a periodical biodegradation phase followed by an aerobic starvation phase in every cycle. Ammonia nitrogen, nitrite, nitrate, total nitrogen and BOD_5_ undergo degradation during a typical cycle of operation (Figure 8). After aeration for 2 h, BOD_5_ was decreased to the lowest of 15 mg L^−1^. The rest of the aeration time of about 3 h for nitrification caused a starvation period of bacteria. Thus, it was likely that microorganism in the SBR were subject to a periodic starvation. After aeration for about 3 hours, due to the degradation of contaminants the aeration intensity decreased for energy saving reasons. The periodic feast-famine conditions facilitated microbial aggregation. Due to the increasing hydrophobicity of the bacteria, a decrease of surface negative charge could be observed [49]. It could be a reason that the most reactors for cultivating aerobic granular sludge were SBR.

The A/O process operated in a plug flow way meaning that an existing feast-famine regime contributed to microbial aggregates. Nevertheless, granules were not formed in the A/O process due to long settling time. Aerobic granules could not be successfully developed if the settling time was not well controlled, even though a periodic feast-famine regime was present [50, 51]. In the SBR, settling time and volume exchange ratio could be easily controlled which acted as the main hydraulic selecting pressures inducing aerobic granulation [52]. Moreover, the mixed liquor flowed into the secondary sedimentation tank for settling in continuous flow process. Part of the settled sludge was returned back to the aeration tank by pumping which could easily destroy the aggregated sludge. Consequently, it was also one reason for an incomplete granulation in the A/O or OD.

## 4. Conclusion

Aerobic granular sludge was successfully cultivated in a lab-scale and a pilot-scale SBR in a WWTP. Subsequently, mature granules with compact structure, an average SVI_30_ of 47.1 mL g^−1^, a diameter of 0.5 mm and a settling velocity of 48 m h^−1^ were achieved in a full-scale SBR. Compared to two continuous flow reactors (A/O and OD) in this plant, different microbial communities were found in the full-scale SBR. The reasons for aerobic granulation in the SBR were related to the composition of raw wastewater and operating mode such as periodic feast-famine, shorter settling time and no return sludge pump.

## Figures and Tables

**Table 1:** A cycle distribution of operation time in lab-scale, pilot-scale, and full-scale SBRs.

	Lab-scale SBR		Pilot-scale SBR		Full-scale SBR		
Operational cycle	0–10 d	11–43 d	0–7 d	7–50 d	0–25 d	25–180 d	180–now
Filling (min)	1	1	40	40	40	40	40
Aerating (min)	120	180	120	120	240	240	240
Settling (min)	60	10	60	20	60	40	50
Discharging (min)	20	20	20	20	30	30	30
Idling (min)	39	29	0	40	0	0	0

**Table 2:** Comparison of sludges in A/O, OD, and full-scale SBRs.

Process	Anaerobic/oxic	Oxidation ditch	Full-scale SBR
Volumetric flow rate (m^3^ d^−1^)	10,000	50,000	50,000
Operating mode	Plug flow (A/O)	Continuous flow	Sequencing batch
Substrate change	Feast-famine	Completely mixed	Feast-famine
COD loading (kg m^3^ d^−1^)	0.54	0.68	0.56
Ammonium loading (kg m^3^ d^−1^)	0.025	0.031	0.022
Settling time (min)	150	150	70–80*
MLSS (mg L^−1^)	5471	5818	3946
SVI_30 _(mL g^−1^)	61.1	60.7	47.1
MLVSS/MLSS	56.3%	57.2%	62.4%
Settling velocity (m h^−1^)	10.4	9.2	42.0
EPS (PS, mg/g VSS) (PN, mg/g VSS)	13.0 263.9	15.2 288.2	20.8 253.8
COD removal efficacy (%)	82.2	81.3	85.0
NH_4_^+^-N removal efficacy (%)	94.3	94.8	95.8
TN removal efficacy (%)	25.3%	22.7%	59.6%

*Settling time in full-scale SBR includes 30 min of effluent withdraw.

*Samples from anaerobic/oxic reactor, oxidation ditch, and full scale sequencing batch reactor were taken at the same time on September 8, 2011.

**Table 3:** Similarity coefficients of microbial communities among the samples.

Lane	1	2	3
1	1.00	0.72	0.37
2	0.72	1.00	0.45
3	0.37	0.45	1.00

**Table 4:** Diversity index (*H*) and number of the bands in DGGE profiles of sludge samples.

Sample	1	2	3
Number of bands	23	24	21
*H*	2.82	2.89	2.70

**Table 5:** Comparison of nucleotide sequences and abundance of sequenced DGGE bands.

Band	Sequence	Accession no.	Closest relative (accession no.)	Identity	Relative abundance of bands (%)		
	(bp)			(%)	1#	2#	3#
4	188	KF234427	Uncultured bacterium clone ASNR-16 (JQ809244)	100	7.0	2.3	4.2
8	188	KF234428	Uncultured Chlorobi bacterium clone ABL17 (JQ906960)	97	1.3	1.9	8.4
10	183	KF234429	Uncultured *Nitrospira* sp. (KC491391)	100	11.5	6.7	ND
11	193	KF234430	Uncultured *Nitrosomonas* sp. clone S12 (AY605680)	99	3.5	3.7	ND
12	187	KF234431	*Flavobacterium* sp. R046 (KC252875)	99	ND	ND	7.1
13	188	KF273869	Uncultured Bacteroidetes bacterium (FJ750465)	100	1.4	6.8	ND
14	193	KF234435	Uncultured beta proteobacterium (AB636023)	100	ND	ND	5.7
16	193	KF234436	Uncultured *Aquabacterium *sp. (JQ288705)	99	ND	ND	12.8
18	193	KF234437	*Leptothrix* sp. (JQ946011)	100	ND	ND	5.7
23	170	KF234438	Uncultured Alphaproteobacteriabacterium (CU925173)	100	ND	ND	1.5
25	193	KF234441	Uncultured bacterium (KC541099)	98	ND	ND	3.6
26	193	KF234444	*Thauera *sp. enrichment culture clone LDC-8 (KF020718)	100	8.4	9.2	10.6
28	196	KF234446	Uncultured proteobacterium (GQ243004)	98	ND	ND	2.6

ND: not detected.

The bands are designed as shown in Figure 4.

The abundance was calculated using BandScan (version 5.0) software.

## Highlights

Aerobic granulation was achieved in a 50,000 m^3^ d^−1^ SBR treating a town's wastewater.
Granular sludge from SBR has more compact structure and excellent settling ability.
Bacterial species were distinct in sludges from continuous flow and SBR in this WWTP.
Raw wastewater composition and SBR operating mode were key factors for granulation.
